# Celastrol exerts anti‐inflammatory effect in liver fibrosis via activation of AMPK‐SIRT3 signalling

**DOI:** 10.1111/jcmm.14805

**Published:** 2019-11-19

**Authors:** Yuqin Wang, Chunling Li, Jingya Gu, Chang Chen, Jiaxin Duanmu, Jing Miao, Wenjuan Yao, Jinhua Tao, Mengjue Tu, Biao Xiong, Lingling Zhao, Zhaoguo Liu

**Affiliations:** ^1^ Department of Pharmacology School of Pharmacy Nantong University Nantong China; ^2^ Department of Chemistry and Chemical Engineering Yancheng Institute of Technology Yancheng China

**Keywords:** AMPK, celastrol, inflammation, liver fibrosis, SIRT3

## Abstract

Celastrol, a pentacyclic tritepene extracted from *Tripterygium Wilfordi* plant, showing potent liver protection effects on several liver‐related diseases. However, the anti‐inflammatory potential of celastrol in liver fibrosis and the detailed mechanisms remain uncovered. This study was to investigate the anti‐inflammatory effect of celastrol in liver fibrosis and to further reveal mechanisms of celastrol‐induced anti‐inflammatory effects with a focus on AMPK‐SIRT3 signalling. Celastrol showed potent ameliorative effects on liver fibrosis both in activated hepatic stellate cells (HSCs) and in fibrotic liver. Celastrol remarkably suppressed inflammation in vivo and inhibited the secretion of inflammatory factors in vitro. Interestingly, celastrol increased SIRT3 promoter activity and SIRT3 expression both in fibrotic liver and in activated HSCs. Furthermore, SIRT3 silencing evidently ameliorated the anti‐inflammatory potential of celastrol. Besides, we found that celastrol could increase the AMPK phosphorylation. Further investigation showed that SIRT3 siRNA decreased SIRT3 expression but had no obvious effect on phosphorylation of AMPK. In addition, inhibition of AMPK by employing compound C (an AMPK inhibitor) or AMPK1α siRNA significantly suppressed SIRT3 expression, suggesting that AMPK was an up‐stream protein of SIRT3 in liver fibrosis. We further found that depletion of AMPK significantly attenuated the inhibitory effect of celastrol on inflammation. Collectively, celastrol attenuated liver fibrosis mainly through inhibition of inflammation by activating AMPK‐SIRT3 signalling, which makes celastrol be a potential candidate compound in treating or protecting against liver fibrosis.

## 1 INTRODUCTION

Compelling evidence linked inflammation to the development of liver fibrosis.^1^, ^2^ Currently, few effective drugs can be used to treat liver fibrosis, and suppressing liver inflammation is an effective strategy to control liver fibrosis.^3^ It is well known that hepatic stellate cells (HSCs) activation is regarded as a critical step in mediating liver fibrosis,^4^ and however, continuous secretion of inflammatory cytokines in liver tissue will lead to the persistent activation of HSCs. Moreover, the activated HSCs itself also secrete inflammatory cytokines that further promote liver fibrosis.^5^, ^6^ Thus, suppressing inflammation and improving the inflammatory microenvironment are crucial for treatment of liver fibrosis.

**Figure 1:**
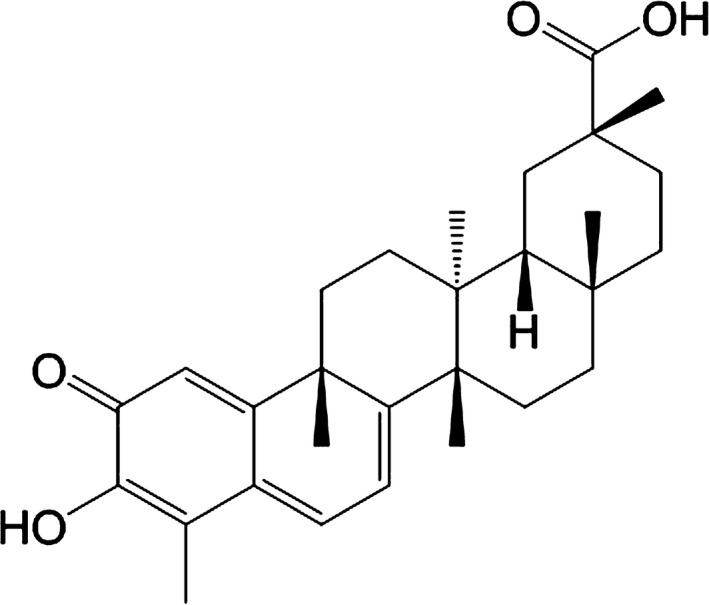
Chemical structure of celastrol

The active ingredients extracted from Traditional Chinese Medicine have become important choices for the treatment of several chronic diseases, including liver fibrosis.^5^, ^7^ Celastrol is a pentacyclic triterpenoid compound^8^, ^9^ (the chemical structure of celastrol is shown in Figure 1), which is isolated from a common clinical used Traditional Chinese Medicine named *Tripterygium wilfordii* Hook F. Celastrol possesses multiple pharmacological effects and showed potent anti‐inflammatory effect against many disease models,^10^, ^11^, ^12^, ^13^ and however, few reports can be seen regarding the anti‐inflammatory potential of celastrol in liver fibrosis. SIR2 is a family of histone deacetylases and is widely distributed in cells with multiple functions.^14^ A total of seven members (SIRT1‐SIRT7) have been identified in mammalian.^15^ SIRT1 is predominantly nuclear and could modify the activity of target proteins through deacetylation, thus contributing to oxidative response and cell cycle control.^16^ SIRT2 is the only sirtuin that is mainly located in the cytoplasm and plays roles in neurological disease, cancers and other diseases.^17^ SIRT3, SIRT4 and SIRT5 are found in the mitochondrial, of which SIRT3 is closely associated with oxidative stress and has been demonstrated involved in many liver‐related diseases.^18^, ^19^ SIRT6 is located in the nucleus with unique and important functions in maintaining cellular homoeostasis.^20^ SIRT7 locates in nucleus and participated in the ribosomal RNA transcription, cell metabolism, cell stress and DNA damage repair.^21^

AMPK has been proved involved in the pathophysiology of liver fibrosis,^22^ and up‐regulation of AMPK phosphorylation facilitated to the attenuation of liver fibrosis.^23^ Interestingly, SIRT3 has been reported as a downstream effector of AMPK in several disease models, and activation of AMPK‐SIRT3 signalling contributes to the improvement of mitochondrial function, thus alleviating the progress of diseases.^24^, ^25^However, in liver fibrosis, whether celastrol regulate AMPK‐SIRT3 signalling remains poorly understood. Moreover, whether activation of AMPK‐SIRT3 signalling contributes to the anti‐inflammatory effect of celastrol remains to be determined. In the current study, the effects of celastrol on liver fibrosis were investigated in vivo and in vitro, and the potential role of AMPK‐SIRT3 signalling in liver fibrosis was assessed for the first time to reveal the underlying mechanisms.

## 2 MATERIALS AND METHODS

### 2.1 Chemicals and reagents

Celastrol (No. PS0048‐0020, purity ≥ 98%) was purchased from Push Bio‐Technology Co., Ltd. (Chengdu, China). Primary antibodies against anti‐rabbit α‐SMA (14395‐1‐AP), α(I) procollagen (14395‐1‐AP), fibronectin (15613‐1‐AP), PPARγ (16643‐1‐AP) and GAPDH (13937‐1‐AP) were purchased from proteintech. Primary antibodies against SIRT3 (#5490), p‐AMPK (#2537) and AMPK (#2532) were purchased from Cell Signalling Technology (Danvers, MA, USA). Primary antibody against PGC‐1α was purchased from Affinity (AF5395). Hydroxyproline examination kit (A030‐2‐1) was purchased from Nanjing Jiancheng Bioengineering Institute. ELISA kits including IL‐6 (H007), IL‐18 (H015), IL‐1β (H002), TNF‐α (H052), IFN‐γ (H025) and IL‐10 (H009) were purchased from Nanjing Jiancheng Bioengineering Institute. Dorsomorphin (Compound C, an AMPK inhibitor) (S7840) was purchased from Selleck. The primers used in real‐time PCR were from GenScript Co. Ltd. MegaTran 1.0 transfection reagent was from OriGene. SIRT3 enzyme activity detection kit (JK50288.2) was purchased from Shanghai Baoman Biotechnology Co., Ltd.

### 2.2 Cell isolation, culture and transfection

Primary rat HSCs were isolated from male Sprague‐Dawley rats weighing 180‐220 g (Shanghai Slac Laboratory Animal) as described previously.^26^ Isolated HSCs were cultured in DMEM with 10% foetal bovine serum and 1% antibiotics, and maintained at 37°C in a humidified incubator of 5% CO_2_ and 95% air. Cell morphology was assessed using an inverted microscope with a Leica Qwin System (Leica). HSCs at passages 2‐4 were used in experiments. SIRT3 siRNA, AMPK1α siRNA and matched negative control siRNA were purchased from RayBiotech. Cell transfection was performed using MegaTran 1.0 transfection reagent. The detail protocol was according to previously reported. The transfection efficiency was confirmed by Western blot analysis.

### 2.3 Cell viability and cytotoxicity assays

Primary activated HSCs were seeded in 96‐well plates, cultured in DMEM supplemented with 10% FBS for 24 hours and then treated with DMSO or celastrol at indicated concentrations for 12 and 24 hours. After treatment, 3‐(4,5‐dimethylthiazol‐2‐yl)‐5‐(3‐carboxymetho‐xyphenyl)‐2‐(4‐sulfo‐phenyl)‐2H‐tetrazolium (MTS; Sigma) solution (5 mg/mL) was added (10 μL/well), and the cells were further incubated for 3 hours at 37°C. The spectrophotometric absorbance at 490 nm was measured by a SPECTRA‐max^™^ microplate spectrophotometer (Molecular Devices). Five duplicate wells were set‐up for each group. For cytotoxicity assay, lactate dehydrogenase (LDH) activity in culture medium was determined with a LDH release assay kit according to the protocol.

### 2.4 Animals and experimental procedures

All experimental procedures were approved by the institutional and local committee on the care and use of animals of Nantong University, and all animals received human care in strict accordance with the National Institutes of Health guidelines. Male Sprague‐Dawley rats (180‐220 g) were purchased from Shanghai Slac Laboratory Animal. Liver fibrosis was induced by a mixture of carbon tetrachloride (CCl_4_; 1 mL/kg) and olive oil (1:1 [v/v]). A total of 40 male rats were randomly divided into five groups, eight rats per group. They were given administration of olive oil (vehicle control; group 1), CCl_4_ (model; group 2), CCl_4_+0.25 mg/kg celastrol (group 3), CCl_4_+0.5 mg/kg celastrol (group 4) and CCl_4_+1 mg/kg celastrol (group 5) (the dose of celastrol referred to Tang et al^12^ and Cheng et al^27^). During the experiment, Groups 2‐5 were given CCl_4_ injection every other day for 8 weeks. The test compound celastrol was dissolved and diluted to the specified concentration by using olive oil. Celastrol was given to rats by i.p. once daily during weeks 5‐8. Groups 1 and 2 were similarly treated, i.p. injection with the same volume of olive oil. At the end of experiment, rats were weighted and anaesthetized by giving 50 mg/kg pentobarbital (i.p. injection), and blood was collected from common carotid arteries by using arterial intubation method in rats and then isolated the livers to calculate liver/body weight ratio. Meanwhile, a small piece of liver was cut and fixed by using 10% formalin to conduct histopathological and immunohistochemical studies. The rest liver was cut into pieces and frozen with liquid nitrogen rapidly to extract total RNA and hepatic proteins.

### 2.5 Liver histopathology

Harvested liver tissues were fixed in 10% neutral buffered formalin and embedded in paraffin. Liver slices of 5 μm thick were prepared and stained with haematoxylin and eosin and masson's trichrome stain by using standard methods. For sirius red collagen staining, thin sections were deparaffinized and stained with picro‐sirius red for 1 hour at room temperature. After washes, sections on the slides were dehydrated in 100% ethanol and in xylene, and then they were mounted in Permount. Photographs were taken in a blinded fashion at random fields. Representative views of liver sections were shown.

### 2.6 Biochemical analyses

Levels of alanine aminotransferase (ALT), aspartate aminotransferase (AST) and alkaline phosphatase (ALP) in serum samples were evaluated using enzyme‐linked immunosorbent assay methods according to the kit protocols (Nanjing Jiancheng Bioengineering Institute). Experiments were performed in triplicate.

### 2.7 Real‐time PCR

Total RNA was extracted from rat liver samples or rat HSCs using Trizol reagent (Biouniquer Technology Co., Ltd.) and then subjected to reverse transcription to cDNA using the kits provided by TaKaRa Biotechnology Co., Ltd. according to the protocol. Amplification kit was purchased from Bio‐Rad Laboratories. GAPDH was used as the invariant control. Results were from triplicate experiments. The following primers of genes (Keygen) were used: α‐SMA: (Forward) 5′‐CCGACCGAATGCAGAAGGA‐3′, (Reverse) 5′‐ACAGAGTATTTGCGCTCCG GA‐3′; α(I)‐procollagen: (Forward) 5′‐CCTCAAGGGCTCCAACG AG‐3′, (Reverse) 5′‐TCAATCACTGTCTTGCCCCA‐3′; Fibronectin: (Forward) 5′‐TGTCACCCACC ACCTTGA‐3′, (Reverse) 5′‐CTGATTGTTCTTCAGTGCGA‐3′; PPARγ: (Forward) 5′‐ATTCTGGCCCACCAACTTCGG‐3′, (Reverse) 5′‐TGGAAGCCTGATGCTTT ATCCCCA‐3′; Rat SIRT1: (Forward) 5′‐CACCAGAAAGAACTTCACCACCAGA ‐3′, (Reverse) 5′‐ACCATCAAGCCGCCTACTAATCTG‐3′; Rat SIRT2: (Forward) 5′‐AGGGACAAGGAGCAGGGTTC‐3′, (Reverse) 5′‐GAAGAGAGACAGCGGCA GGAC‐3′; Rat SIRT3: (Forward) 5′‐GAGGTTCTTGCTGCATGTGGTTG‐3′, (Reverse) 5′‐AGTTTCCCGCTGCACAAGGTC‐3′; Rat SIRT4: (Forward) 5′‐TT GTGCCAGCAAGTCCTCCTC‐3′, (Reverse) 5′‐GTCTCTTGGAAAGGGTGATGA AGC‐3′; Rat SIRT5: (Forward) 5′‐TCCAGCGTCCACACGAAACC‐3′, (Reverse) 5′‐AACACCAGCTCCTGAGATGATGAC‐3′; Rat SIRT6: (Forward) 5′‐GCTGGA GCCCAAGGAGGAATC‐3′, (Reverse) 5′‐AGTAACAAAGTGAGACCACGAGA G‐3′; Rat SIRT7: (Forward) 5′‐GAGCCAACCCTCACCCACATG‐3′, (Reverse) 5′‐ACGC AGGAGGTACAGACTTCAATG‐3′; GAPDH: (Forward) 5′‐GGCCCCTC TGGAAA GCTGTG‐3′, (Reverse) 5′‐CCGCCTGCTTCACCACC TTCT‐3′.

### 2.8 Western blot analyses

RIPA buffer (Beyotime) supplemented with PMSF (Beyotime) was used form protein extraction from liver tissues and HSCs cells. Protein concentration was measured using an Enhanced BCA Protein Assay kit (Beyotime). The protocol of Western blot analysis was according to previously reported.^28^ Representative blots were from three independent experiments.

### 2.9 Enzyme‐linked immunosorbent assay (ELISA)

The levels of interleukin‐6 (IL‐6), IL‐10, IL‐13, IL‐18, IL‐1β, interferon‐γ (IFN‐γ) and tumour necrosis factor‐α (TNF‐α) in liver tissues and HSCs culture supernatant were determined using ELISA kits (Nanjing Jiancheng Bioengineering Institute) according to the standard protocols.^29^ Results were from triplicate experiments.

### 2.10 Luciferase reporter assay

Hepatic stellate cells cells were cotransfected with SIRT3 promoter luciferase fusion plasmid and pRL‐TK reporter plasmid (control reporter) using Lipofectamine 3000 reagent (Invitrogen).^14^ Twenty‐four hours later, cells were stimulated with PDGF‐BB (20 ng/mL) for another 24 hours following treatment with celastrol (20 μmol/L) for 4 hours. Then, the fluorescence intensity was determined using a dual luciferase reporter assay system (Promega, Madison).

### 2.11 SIRT3 enzyme activity assay

SIRT3 enzyme activity in rat's liver was detected using a SIRT3 enzyme activity detection kit (Baomanbio), using colorimetric method according to the protocol, and results were from triplicate experiments.

### 2.12 Statistical analysis

All experimental data were presented as mean ± SD, and results were analysed by using Statistical Package for Social Sciences (SPSS) (IBM Incorporation) version 16.0 software. The significance of difference was determined by one‐way ANOVA with the post hoc Dunnett's test. Values of *P* < .05 were considered statistically significant.

## 3 RESULTS

### 3.1 Celastrol ameliorated CCl_4_‐caused liver injury and fibrosis in rats

**Figure 2:**
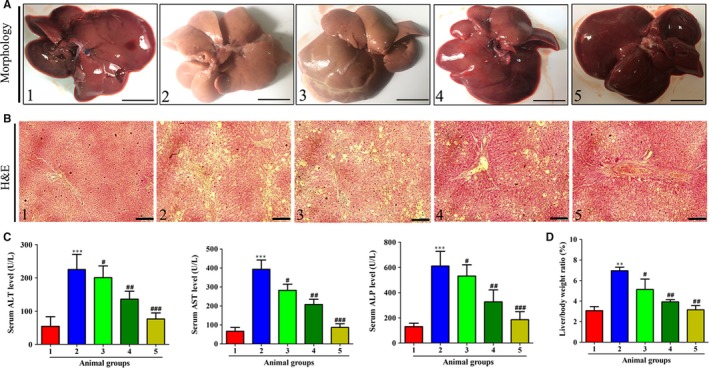
Celastrol ameliorated CCl4‐caused liver injury in rats. Rats were grouped as follows: 1. control group (no CCl4, no treatment); 2. model group (with CCl4, no treatment); 3. celastrol (0.25 mg/kg) and CCl4 treated group; 4. celastrol (0.5 mg/kg) and CCl4 treated group; and 5. celastrol (1 mg/kg) and CCl4 treated group. A, Gross examination of rat livers. Representative photographs are shown (scale bar: 1 cm). B, Liver sections were stained with H&E for histological examination. Representative photographs are shown (scale bar: 100 μm). C, Determination of the serum levels of ALT, AST and ALP. D, The ratio of liver/body weight (%). Data are expressed as Mean ± SD, and each group had eight rats. Significance: **P < .01 and ***P < .001 vs the control group; #*P* < .05, ##*P* < .01 and ###*P* < .001 vs the model group

**Figure 3:**
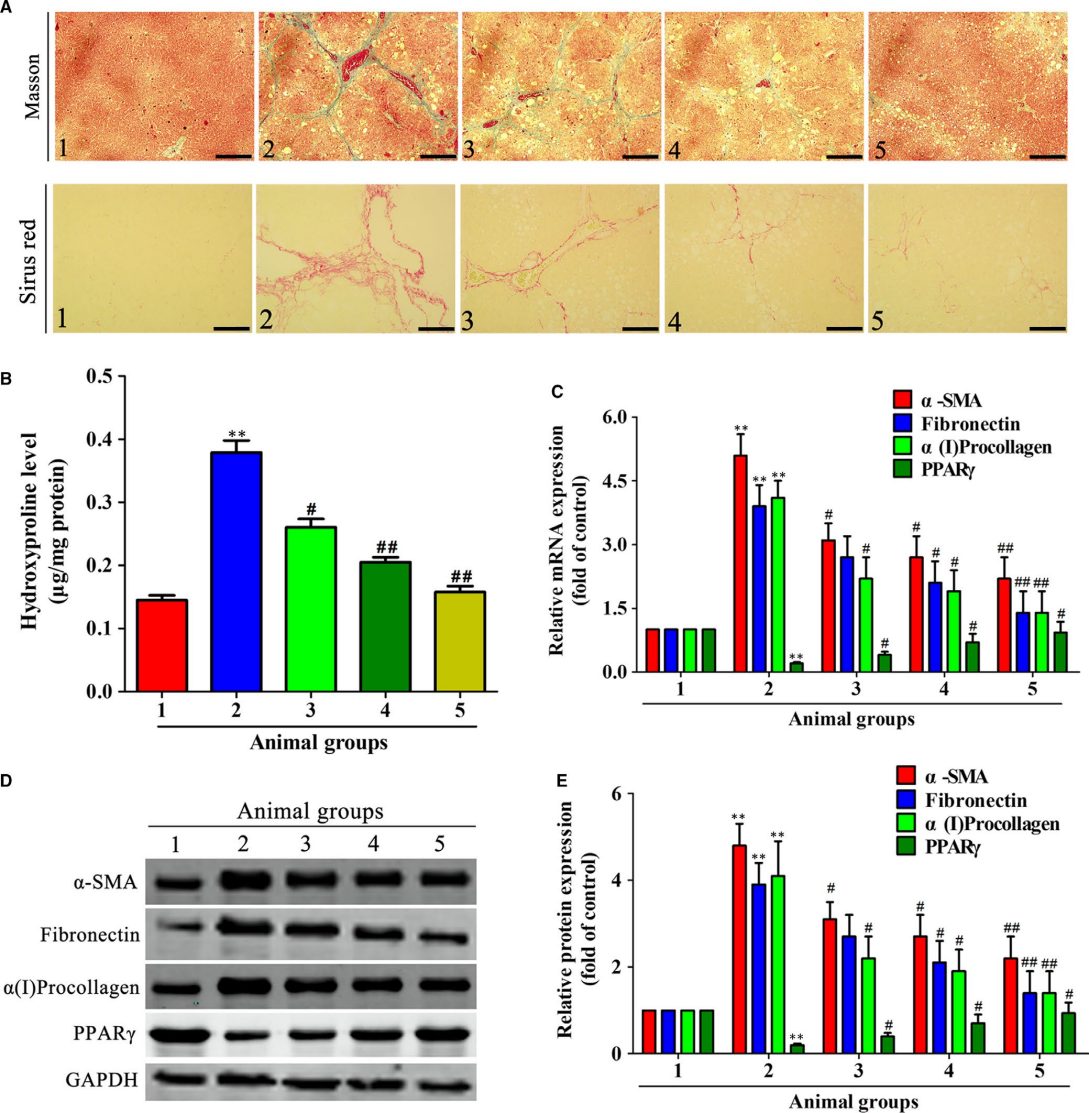
Celastrol attenuated CCl4‐induced liver fibrosis in rats. Rats were grouped as follows: 1. control group (no CCl4, no treatment); 2. model group (with CCl4, no treatment); 3. celastrol (0.25 mg/kg) and CCl4 treated group; 4. celastrol (0.5 mg/kg) and CCl4 treated group; and 5. celastrol (1 mg/kg) and CCl4 treated group. A, Liver sections were stained with masson (scale bar: 100 μm) and sirus red (scale bar: 50 μm) reagents, respectively, and representative photographs are shown (magnification, 200×). B, Measurement of hydroxyproline levels in liver homogenate. C, Real‐time PCR analyses of a‐SMA, fibronectin, α(I)procollagen and PPARγ in liver tissues. GAPDH was used as the invariant control. D, Western blot analyses of liver proteins with densitometry. Representative blots were from three independent experiments. Significance: *P < .05 and **P < .01 vs the control group; #*P* < .05 and ##*P* < .01 vs the model group

We initially evaluated the effect of celastrol on CCl_4_‐induced hepatic injury in vivo. Rats received CCl_4_ injection resulted in obvious changes in liver morphology, and celastrol improved the morphology changes in livers (Figure 2A). Celastrol remarkably improved the CCl_4_‐induced liver pathology, as evidenced by ameliorated state of hepatic steatosis, necrosis and fibrotic septa (Figure 2B). Celastrol remarkably decreased the serum levels of ALT, AST and ALP compared with the model group (Figure 2C). The high ratio of liver/body weight induced by CCl_4_ was reduced by treatment with celastrol (Figure 1C). As continuous administration of CCl_4_ could lead to liver fibrosis,^30^ therefore, we then studied the effect of celastrol on CCl_4_‐induced liver fibrosis in rats. Celastrol decreased the collagen deposition caused by CCl_4_ injection (Figure 3A). Hepatic hydroxyproline examination further showed that celastrol decreased the CCl_4_‐induced collagen production (Figure 3B). Besides, celastrol inhibited the mRNA and protein expressions of α‐SMA, fibronectin and α(I) procollagen but rescued the decreased PPARγ expression in rat fibrotic liver (Figure 3C‐E). Taken together, celastrol attenuated CCl_4_‐induced liver injury and fibrosis in rats.

### 3.2 Celastrol suppressed inflammation in rat fibrotic liver

**Figure 4:**
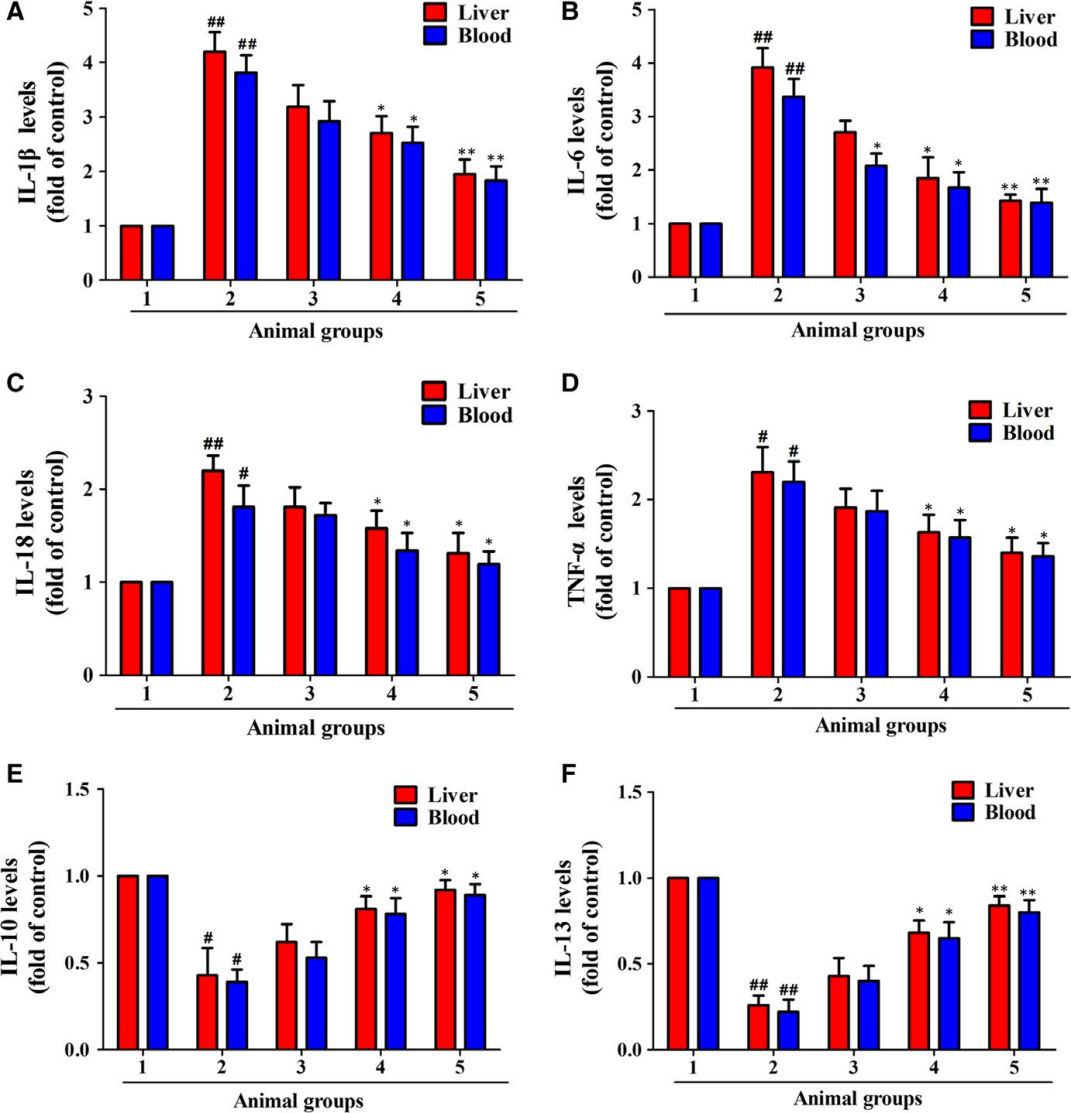
Celastrol suppressed inflammation in rat fibrotic liver. Rats were grouped as follows: 1. control group (no CCl4, no treatment); 2. model group (with CCl4, no treatment); 3. celastrol (0.25 mg/kg) and CCl4 treated group; 4. celastrol (0.5 mg/kg) and CCl4 treated group; and 5. celastrol (1 mg/kg) and CCl4 treated group. A‐D, ELISA analyses of pro‐inflammatory factors IL‐6, IL‐18, IL‐1β, TNF‐α and IL‐10 in liver tissues and serum. E‐F, ELISA analyses of anti‐inflammatory factors IL‐10 and IL‐13 in liver tissues and blood. Data are expressed as mean ± SD, and the experiments were repeated three times. Significance: #*P* < .05 vs the control group, ##*P* < .01 vs the model group; *P < .05 and **P < .01 vs the model group

Inflammation is directly linked to liver fibrosis,^1^ and suppressing inflammation can effectively control liver fibrosis. We herein studied the effects of celastrol on the levels of inflammatory factors in liver and serum of rats. As shown in Figure 4, compared with the control group, CCl_4_ injection significantly elevated the levels of pro‐inflammatory factors IL‐1β, IL‐6, IL‐18 and TNF‐α (Figure 4A‐D), and decreased the levels of anti‐inflammatory factors IL‐10 and IL‐13 (Figure 4E‐F). However, treatment with celastrol remarkably suppressed the levels of IL‐1β, IL‐6, IL‐18 and TNF‐α while increased the levels of IL‐10 and IL‐13 in fibrotic livers and serum (Figure 4A‐F), showing potent anti‐inflammatory property. Overall, celastrol suppressed the inflammation in rat fibrotic liver.

### 3.3 Celastrol attenuated liver fibrosis and inflammation in activated HSCs in vitro

**Figure 5:**
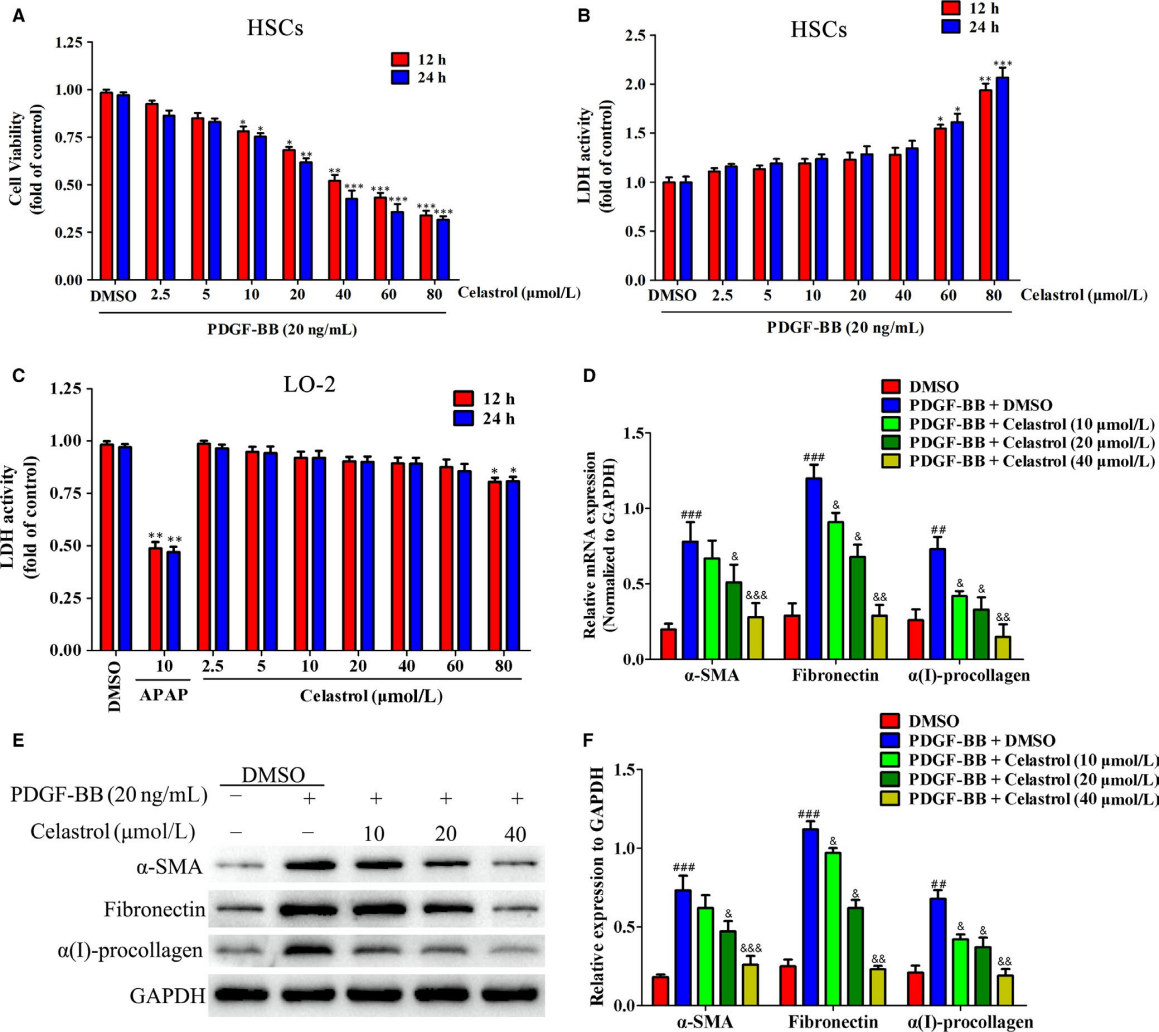
Celastrol inhibited liver fibrosis in activated hepatic stellate cell (HSCs) in vitro. HSCs were treated with DMSO (0.02%, w/v), PDGF‐BB (20 ng/mL), and the indicated concentrations of celastrol for 24 h. A, The effect of celastrol on cell viability of activated HSCs in vitro. B, The toxicity of celastrol on activated HSCs was examined by LDH activity assay. C, The toxicity of celastrol on LO2 was examined by LDH activity assay. D, RT‐PCR analyses of α‐SMA, fibronectin and α1(I)‐procollagen in HSCs treated with 20 ng/mL PDGF‐BB and celastrol (10, 20 and 40 µmol/L) for 24 h compared with vehicles treated cells (Control), GAPDH was used as an invariant control for equal loading. E, Western blot analyses of α‐SMA, fibronectin and α1(I)‐procollagen in HSCs treated with 20 ng/mL PDGF‐BB and celastrol (10, 20 and 40 µmol/L) for 24 h compared with vehicles treated cells (Control), GAPDH was used as an invariant control for equal loading. DF, Quantified Western blot results of cell proteins. Data are expressed as mean ± SD, and the experiments were repeated three times. Significance: *P < .05, **P < .01 and ***P < .001 vs the DMSO group; &*P* < .05, &&*P* < .01 and &&&*P* < .001 vs the PDGF‐BB + DMSO group

**Figure 6:**
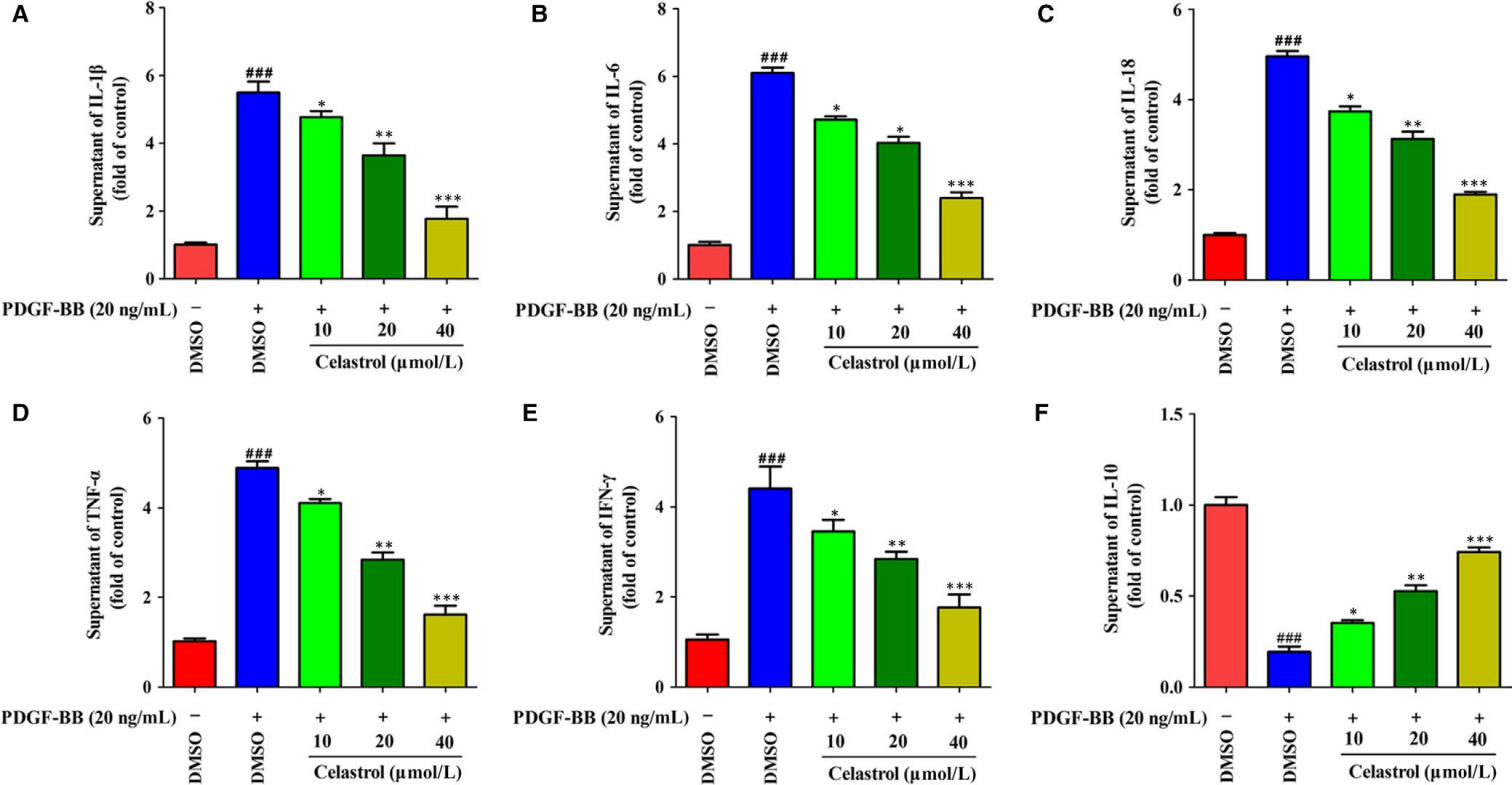
Celastrol suppressed inflammation in activated HSCs in vitro. HSCs were treated with DMSO (0.02%, w/v), PDGF‐BB (20 ng/mL), and the indicated concentrations of celastrol for 24 h. A‐E, ELISA measurement of pro‐inflammatory factors IL‐1β, IL‐6, IL‐18, TNF‐α, IFN‐γ levels in supernatant of activated HSCs, respectively. F, ELISA measurement of anti‐inflammatory factor IL‐10 level in supernatant of activated HSCs. Data are expressed as mean ± SD, and the experiments were repeated three times. Significance: ###*P* < .001 vs the DMSO group; *P < .05, **P < .01 and ***P < .001 vs the PDGF‐BB + DMSO group

As blocking, the HSCs activation helps to the control of liver fibrosis.^31^ Thus, we further studied the effects of celastrol on liver fibrosis and inflammation in activated HSCs. Celastrol inhibited the viability of activated HSCs in a concentration‐dependent manner and resulted in a significant inhibitory effect at 10 μmol/L (Figure 5A). Moreover, celastrol showed toxicity at 60 μmol/L in activated HSCs (*P* < .05) (Figure 5B). We further examined the toxicity of celastrol on normal liver cells LO2. Celastrol showed no toxicity on LO2 below 80 μmol/L (Figure 5C). We next investigated the effect of celastrol on expressions of α‐SMA, fibronectin and α(I) procollagen in activated HSCs. Celastrol notably inhibited the mRNA and protein expressions of α‐SMA, fibronectin and α(I) procollagen in activated HSCs (Figure 5D‐F). The effect of celastrol on secretion of inflammation factors in activated HSCs was further examined. Celastrol suppressed the secretion of pro‐inflammatory factors IL‐1β, IL‐6, IL‐18, TNF‐α and IFN‐γ (Figure 6A‐E) while increased the secretion of IL‐10 (Figure 6F) in activated HSCs. Collectively, celastrol inhibited liver fibrosis and inflammation in activated HSCs.

### 3.4 Celastrol promoted SIRT3 expression and activity in activated HSCs and rat fibrotic livers

**Figure 7:**
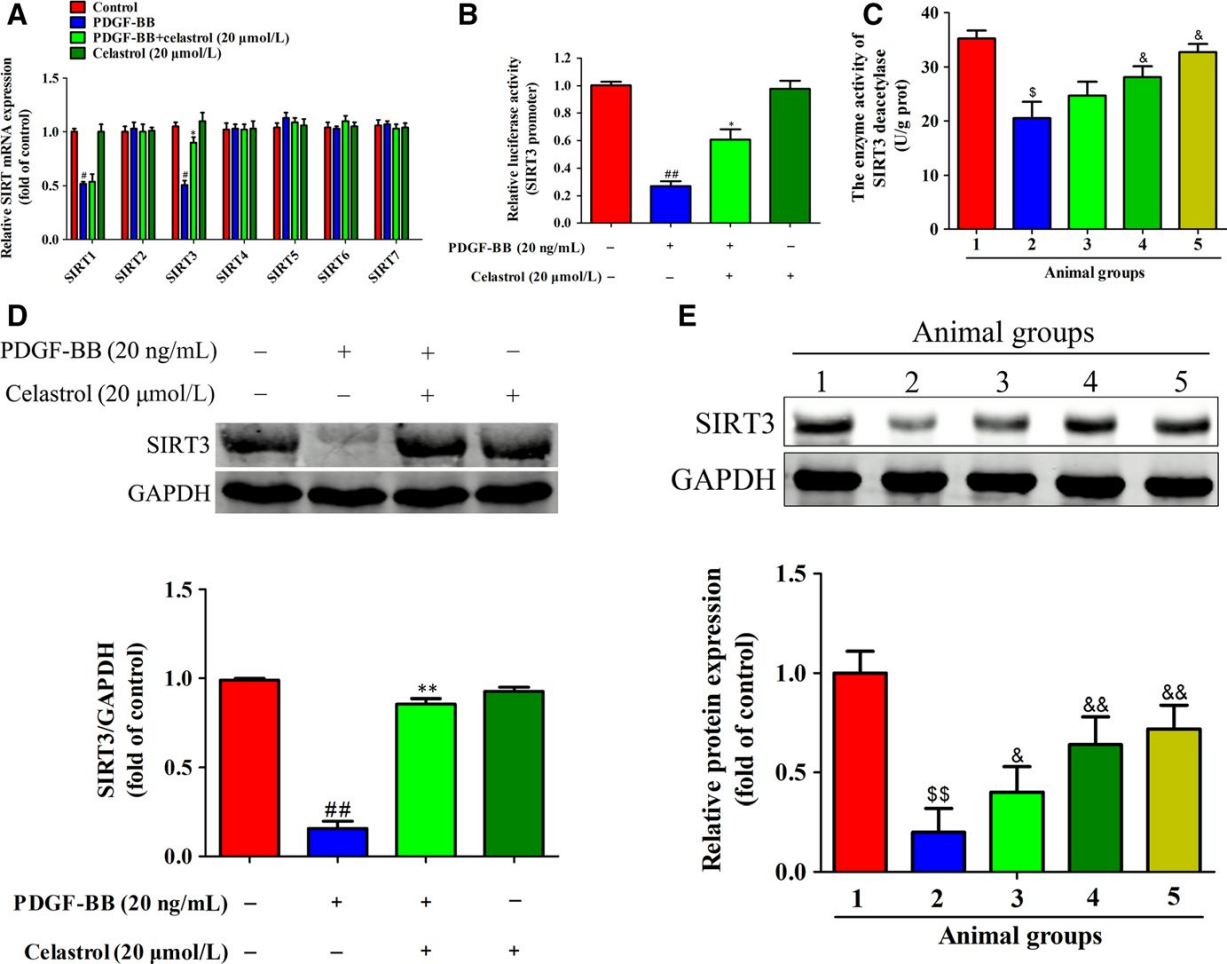
Celastrol promoted SIRT3 expression in activated HSCs and rat fibrotic livers. A, Quantification of SIR2 family (SIRT1‐SIRT7) mRNA expression was assessed by real‐time PCR. B, SIRT3 promoter luciferase activity was evaluated with a dual luciferase reporter assay system. C, The effect of celastrol on the enzyme activity of SIRT3 deacetylase in rat fibrotic livers. D, Quantification of SIRT3 protein expression was assessed by Western blots in vitro. ED, Quantification of SIRT3 protein expression in rat fibrotic liver was assessed by Western blots. Rats were grouped as follows: 1. control group (no CCl4, no treatment); 2. model group (with CCl4, no treatment); 3. celastrol (0.25 mg/kg) and CCl4 treated group; 4. celastrol (0.5 mg/kg) and CCl4 treated group; and 5. celastrol (1 mg/kg) and CCl4 treated group. Data are expressed as mean ± SD, and the experiments were repeated three times. Significance: #*P* < .05 and ##*P* < .01 vs the DMSO group; *P < .05 and **P < .01 vs the PDGF‐BB group; $*P* < .05 and $$*P* < .01 vs the control group; &*P* < .05 and &&*P* < .01 vs the control group

As sirtuin family participated in regulation of liver fibrosis,^32^ however, a total of senven sirtuin members (SIRT1‐SIRT7) in sirtuin family, thus, which member played a role in liver fibrosis, should be firtstly determined. Of all the sirtuin members, we found only SIRT1 and SIRT3 showed decrease in mRNA expressions in activated HSCs (Figure 7A). Interestingly, treatment with celastrol increased SIRT3 but not SIRT1 mRNA expression (Figure 7A), suggesting that SIRT3 participated in the attenuation effect of celastrol on liver fibrosis. Besides, celastrol increased the activity of SIRT3 promoter in activated HSCs (Figure 7B). Celastrol notably increased the SIRT3 deacetylase activity in rat fibrotic livers (Figure 7C). Moreover, celastrol promoted SIRT3 protein expression both in activated HSCs (Figure 7D) and in rat fibrotic livers (Figure 7E). Altogether, celastrol increased SIRT3 expression in vivo and in vitro.

### 3.5 Depletion of SIRT3 ameliorated the anti‐inflammatory effect of celastrol in activated HSCs

**Figure 8:**
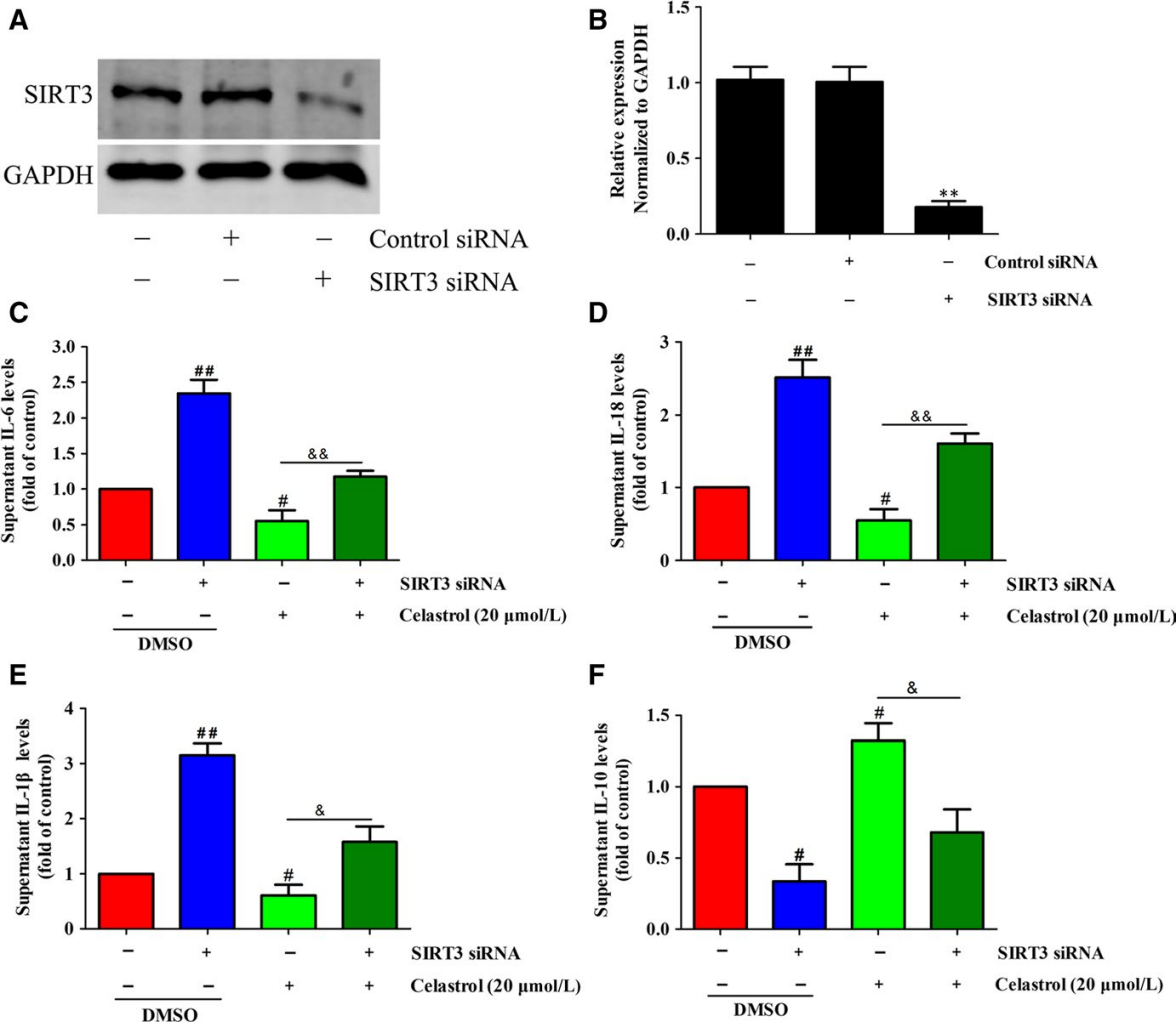
Depletion of SIRT3 ameliorated the anti‐inflammatory effect of celastrol in activated HSCs. HSCs were transfected with SIRT3 siRNA or control siRNA and then were treated with the indicated concentration of celastrol for 24 h. A, The transfection efficiency was confirmed by Western blot analysis. B, Quantitation of the result of Western blot. C‐F, ELISA measurement of IL‐6, IL‐18 and IL‐1β levels in supernatant of HSCs treated with 20 µmol/L celastrol for 24 h following SIRT3 siRNA treatment. Data are expressed as mean ± SD, and the experiments were repeated three times. Significance: **P < .01 vs the control siRNA group; #*P* < .05 and ##*P* < .01 vs the control (DMSO) group; &*P* < .05 and &&*P* < .01 vs the celastrol (20 μmol/L) alone treated group

As celastrol increased SIRT3 expression, then, we wonder whether SIRT3 enhancement was essential for the inflammation inhibition effect of celastrol in activated HSCs. Thus, the effects of SIRT3 silencing on the anti‐inflammatory effect of celastrol were studied. The protein expression of SIRT3 was reduced significantly after transfected with SIRT3 siRNA (Figure 8A,B), indicating that SIRT3 was successfully interfered. Compared with celastrol treated alone group, SIRT3 siRNA decreased the inhibitory capacity of celastrol on IL‐6, IL‐18 and IL‐1β in activated HSCs (Figure 8C‐E). In addition, SIRT3 silencing reduced the promotion effect of celastrol on IL‐10 (Figure 8F). Overall, SIRT3 is crucial for the anti‐inflammatory role of celastrol in liver fibrosis.

### 3.6 Activation of AMPK signalling is essential for induction of SIRT3 enhancement by celastrol in activated HSCs

**Figure 9:**
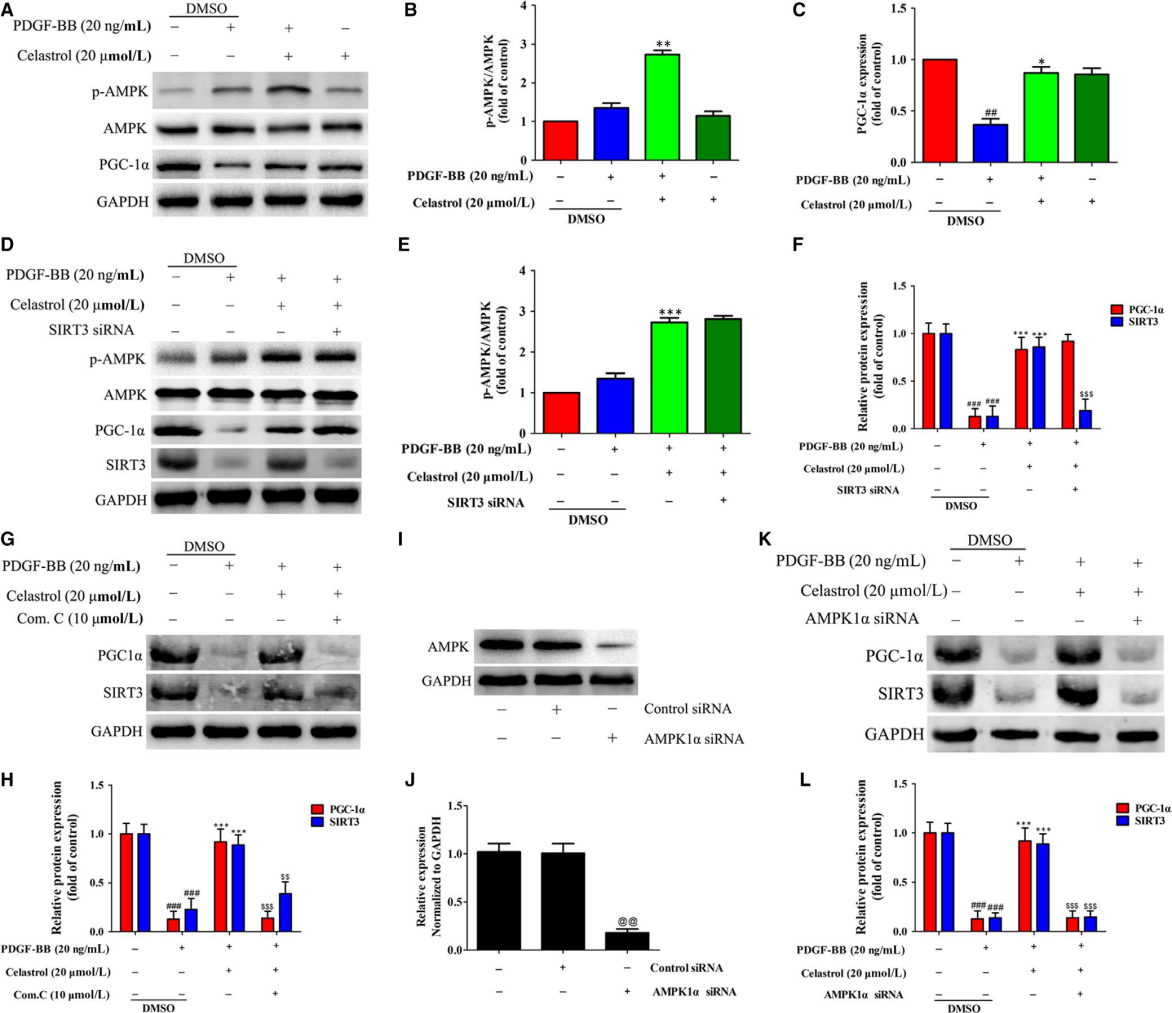
Activation of AMPK signalling is essential for induction of SIRT3 enhancement by celastrol in activated HSCs. A, Western blot analyses of p‐AMPK, AMPK and PGC‐1α in HSCs treated with 20 ng/mL PDGF‐BB and catalpol (20 µmol/L) for 24 h. B, Quantitation of AMPK phosphorylation. C, Quantitation of the PGC‐1α expression. D, The effect of SIRT3 siRNA on expressions of p‐AMPK, AMPK, PGC‐1α and SIRT3 were examined by Western blot. E, Quantitation of AMPK phosphorylation. F, Quantitation of the PGC‐1α and SIRT3 expressions. G, The effect of Compound C (an AMPK inhibitor) on expressions of PGC‐1α and SIRT3 were examined by Western blot. H. Quantitation of the PGC‐1α and SIRT3 expressions. I, The transfection efficiency was confirmed by Western blot analysis. J, Quantitation of the result of Western blot. K, The effect of AMPK1α siRNA on expressions of PGC‐1α and SIRT3 were examined by Western blot. L, Quantitation of the PGC‐1α and SIRT3 expressions. Data are expressed as mean ± SD, and the experiments were repeated three times. Significance: ##*P* < .01 and ###*P* < .001 vs the control (DMSO) group; *P < .05, **P < .01 and ***P < .001 vs the PDGF‐BB + DMSO group; $$*P* < .01 and $$$*P* < .001 vs the PDGF‐BB+ celastrol (20 μmol/L) group

As celastrol increased SIRT3 expression in activated HSCs, however, how celastrol increase SIRT3 expression remains to be determined. AMPK‐PGC‐1α signalling involved in regulating SIRT3.^33^, ^34^ Thus, whether activation of AMPK‐PGC‐1α participated in the regulation of SIRT3 in liver fibrosis was further investigated. Phospho‐AMPK/AMPK ratio was slightly increased by PDGF‐BB stimulation but showed no statistically difference compared with the control group (Figure 9A,B). Celastrol further increased AMPK phosphorylation and effectively increased PGC‐1α expression in activated HSCs (Figure 9A‐C), showing celastrol activated AMPK‐PGC‐1α signalling in activated HSCs. To further study the critical role of SIRT3 in mediating the protection effect of celastrol on liver fibrosis, we then examined the effect of SIRT3 depletion on AMPK‐PGC‐1α signalling in activated HSCs. Interestingly, we found that SIRT3 siRNA suppressed SIRT3 expression (compared with PDGF‐BB+ celastrol group) without causing significant changes in AMPK phosphorylation and PGC‐1α expression (Figure 9D‐F), suggesting that SIRT3 acts as the down‐stream target of the AMPK‐PGC‐1α signalling in liver fibrosis. To further reveal the mechanisms underlying the SIRT3 enhancement induced by celastrol, inhibition of AMPK was employed by using AMPK selective inhibitor Compound C. Compound C treatment significantly inhibited the protein levels of PGC‐1α and SIRT3 (compared with PDGF‐BB+ celastrol group) (Figure 9G,H). We further silenced AMPK by employing AMPK1α siRNA. AMPK1α siRNA effectively reduced AMPK expression, showing that AMPK was successfully interfered (Figure 9I,J). AMPK1α siRNA significantly inhibited the protein levels of PGC‐1α and SIRT3 (compared with PDGF‐BB + celastrol group) (Figure 9K,L). Taken together, activation of AMPK signalling is crucial for induction of SIRT3 enhancement by celastrol in activated HSCs.

### 3.7 Inhibition AMPK signalling decreased the anti‐inflammatory ability of celastrol in activated HSCs

**Figure 10:**
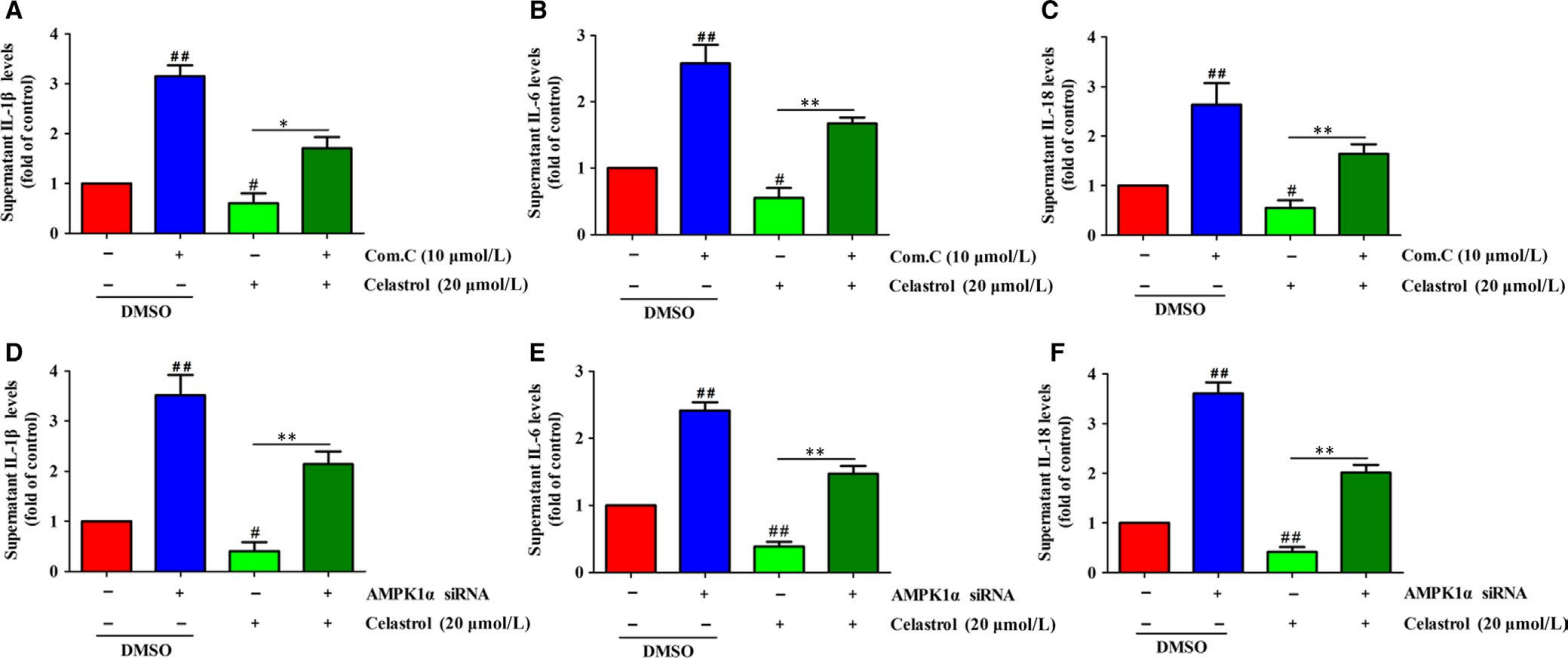
Inhibition AMPK signalling decreased the anti‐inflammatory ability of celastrol in activated HSCs. HSCs were transfected with AMPK1α siRNA or treatment with Compound C and then were treated with the indicated concentration of celastrol for 24 h. A‐C, ELISA measurement of IL‐1β, IL‐6 and IL‐18 levels in supernatant of HSCs treated with 20 µmol/L celastrol for 24 h following Compound C (10 µmol/L) treatment. D‐F, ELISA measurement of IL‐1β, IL‐6 and IL‐18 levels in supernatant of HSCs treated with 20 µmol/L celastrol for 24 h following AMPK1α siRNA treatment. Data are expressed as mean ± SD, and the experiments were repeated three times. Significance: ##*P* < .01 vs the control (DMSO) group; *P < .05 and **P < .01 vs the celastrol (20 μmol/L) alone treated group

The above data showed that celastrol could activate AMPK signalling in liver fibrosis, and thus, whether activation of AMPK signalling is essential for the inhibition of inflammation of celastrol in liver fibrosis was further clarified. As expected, celastrol remarkably suppressed the secretion of IL‐6, IL‐18 and IL‐1β compared with those of the untreated group (Figure 10A‐C). Treatment with Compound C effectively attenuated the anti‐inflammatory effect of celastrol (Figure 10A‐C). The same results were obtained by treatment with AMPK1α siRNA (Figure 10D‐F). Altogether, activation of AMPK signalling is required for the anti‐inflammatory effect of celastrol in activated HSCs.

## 4 DISCUSSION

In this study, we proved that the anti‐fibrotic effect of celastrol was attributed to its anti‐inflammatory effect, and such anti‐inflammatory role of celastrol was dependent on activation of AMPK‐SIRT3 signalling. In particular, our results showed that celastrol exerted its anti‐fibrotic effect by suppressing inflammation. Interestingly, celastrol was identified as a potent activator of AMPK‐SIRT3 signalling, and AMPK or SIRT3 depletion impaired celastrol‐induced anti‐inflammatory effects. As far as we know, few reports can be seen on assessing the anti‐fibrotic role of celastrol *via* AMPK‐SIRT3 activation‐dependent anti‐inflammatory effect.

Accumulating evidence shows that large amounts of inflammatory cytokines gather around the fibrotic liver and makes liver in an inflammatory microenvironment.^5^ Therefore, effective suppression of inflammation and improvement of inflammatory microenvironment are critical to control the progression of liver fibrosis. Currently, anti‐inflammatory drugs have been widely used to control liver inflammation in clinical, of which, remarkable achievements has been made using natural products to prevention and treatment of liver fibrosis in experimental and clinical research.^7^, ^35^ *Tripterygium wilfordii Hook F* (TwHF), a Chinese medicinal plant, shows potent anti‐inflammatory effect, especially in rheumatoid arthritis (RA). Celastrol is an active ingredient of TwHF, which is clinically used to treat the immune diseases.^36^ Currently, celastrol is documented as a potent agent for treating inflammatory disorders. Celastrol attenuated the inflammatory response of the spinal cord after spinal cord injury by reducing the release of TNF‐α, IL‐1β and IL‐18 inflammatory factors, and increasing the release of IL‐10 cytokines.^37^ Besides, celastrol showed potent anti‐inflammatory effect against diet‐induced obesity mainly by regulating macrophage polarization.^38^ In parallel with these existing studies, our results further showing that celastrol had strong anti‐inflammatory effect in liver fibrosis both in vivo and in vitro, suggesting that inflammation intervention was an effective strategy helps to the control of liver fibrosis.

As SIRT3 mainly located in mitochondria and is closely associated with oxidative stress, thus, the existing research mainly focus on its role on oxidative stress.^39^, ^40^ Actually, SIRT3 can also regulate inflammation in various disease models. Boniakowski, et al^41^ showed that under prediabetic conditions, SIRT3 was decreased in wound macrophages and resulted in dysregulated inflammation, indicating that SIRT3 is essential for controlling inflammation in diabetic wounds. Another study reported that SIRT3 could diminish inflammation and mitigated endotoxin‐induced acute lung injury (ALI),^42^ suggesting that the induction/activation of SIRT3 may serve as a new therapeutic strategy in ALI. Besides, SIRT3 deficiency aggravated cisplatin‐induced nephrotoxicity mainly by increasing renal inflammation.^43^ The current study showed that SIRT3 expression was increased by celastrol treatment in liver fibrosis, showing that SIRT3 is essential for the liver protection effect of celastrol against liver fibrosis. Moreover, further study demonstrated that SIRT3 disruption suppressed the secretion and expressions of inflammatory cytokines and improved the inflammatory microenvironment in liver fibrosis, indicating that SIRT3 intervene attenuated the anti‐inflammatory effect of celastrol. Another important point is that TGF‐β is considered as the key promoting factor in liver fibrosis,^44^ as celastrol could up‐regulate SIRT3, whether there is a regulatory role between SIRT3 and TGF‐β? Previous study showed that SIRT3 could deacetylate and activate glycogen synthase kinase 3β (GSK‐3β) to positively regulate its activity, which phosphorylated β‐catenin and Smad to block TGF‐β1 signalling and suppress tissue fibrosis.^45^ Therefore, in the future study, the effect of celastrol on GSK‐3β, phosphorylation of β‐catenin and Smad will be investigated to clarify the regulatory role between SIRT3 and TGF‐β.

AMPK is involved in the development of many disease models including liver fibrosis and activation of AMPK inhibited NF‐κB signalling to alleviate liver inflammation and fibrosis.^45^ Liver fibrosis can also be ameliorated by AMPK phosphorylation and blockade of mTOR‐dependent cascades.^46^ Consistent with this study, the present study showed that celastrol evidently increased the expression of phosphorylation of AMPK in liver fibrosis and inhibited AMPK by using selective inhibitor Compound C attenuated the anti‐inflammatory effect of celastrol, suggesting that activation of AMPK might contribute to the anti‐inflammatory effect of celastrol under liver fibrosis conditions. Another important point in our study was that PDGF‐BB stimuli slightly increased the AMPK phosphorylation, but celastrol treatment significantly increased it, and we guess that it may be due to PDGF‐BB stimuli HSCs formed a pathological environment (namely in vitro liver fibrosis model), and celastrol increased AMPK phosphorylation only in pathological environment but not in normal condition, thus celastrol alone (without PDGF‐BB stimulate) had no obvious effect on AMPK phosphorylation. Further experiments are needed to confirm our hypotheses. Noteworthy, PGC‐1α served as an important down‐stream protein of AMPK. Under inflammatory condition, activation of AMPK/PGC‐1α signalling facilitated to the improvement of cerebral ischaemic stroke mainly by suppressing neuroinflammation.^47^ Previously, celastrol was reported to exert antioxidant effects by increasing the AMPK phosphorylation level in the skeletal muscle of diabetic rats.^48^ This study celastrol promoted PGC1α expression in PDGF‐BB‐induced liver fibrosis model, but celastrol alone had no obvious effects on AMPK phosphorylation and PGC1α expression, showing that the expressions of AMPK and PGC1α were regulated by celastrol may only in pathological conditions.

Intriguingly, previous studies showed that SIRT3 could activate AMPK in some disease models. Zhang et al reported that caffeine improved hepatic steatosis partly by activation of SIRT3/AMPK pathway, and SIRT3 silencing decreased the AMPK phosphorylation in the liver.^49^ Another study indicated that SIRT3 could reduce lipid accumulation through AMPK activation in human hepatic cells, and further proved that the activity of SIRT3 deacetylase was required for SIRT3 to activate AMPK.^48^ On the contrary, accumulating evidence suggested that AMPK could regulate SIRT3. Yu, et al found that naringenin ameliorated ischaemia‐reperfusion injury mainly by activation of AMPK‐SIRT3 signalling pathway.^25^ In their study, inhibition of AMPK by AMPK1α siRNA or Compound C (AMPK inhibitor) markedly suppressed SIRT3 levels, and however, SIRT3 siRNA could not significantly change AMPK phosphorylation, showing that SIRT3 was the down‐stream target of AMPK. Therefore, the above studies hinted that a crosstalk may exist between AMPK and SIRT3 signalling. Interestingly, previous studies have shown that celastrol exerted antioxidant effects on skeletal muscle of diabetic rats partly by regulating AMPK‐SIRT3 signalling.^48^ Consistent with the previous study, under pathological conditions of liver fibrosis, inhibition of AMPK evidently decreased SIRT3 expression and reduced inflammatory inhibition effect of celastrol. Nevertheless, SIRT3 depletion remarkably had no obvious effect on AMPK phosphorylation. These data suggested that the interplay between AMPK and SIRT3 participated in the inflammatory inhibition effect of celastrol in liver fibrosis, and SIRT3 was a critical down‐stream target of AMPK in liver fibrosis. In summary, we confirm that celastrol activated AMPK‐SIRT3 signalling, and activation of AMPK‐SIRT3 signalling facilitated to the anti‐inflammatory effect of celastrol against liver fibrosis.

## CONFLICT OF INTEREST

The authors confirm that there are no conflicts of interest.

## AUTHORS' CONTRIBUTIONS

Yuqin Wang, Chunling Li and Jingya Gu carried out the most of the experiments; Chang Chen and Jiaxin Duanmu analysed the data; Lingling Zhao and Zhaoguo Liu designed the study; Yuqin Wang and Zhaoguo Liu wrote the manuscript; Jing Miao, Wenjuan Yao and Jinhua Tao provided the statistical support; Menjue Tu and Biao Xiong contributed to the critical revision of article. All authors have read the manuscript and approved the final version.

## Data Availability

The data that support the findings of this study are available from the corresponding author upon reasonable request.
